# Extracellular Vesicles (Exosomes) as Immunosuppressive Mediating Variables in Tumor and Chronic Inflammatory Microenvironments

**DOI:** 10.3390/cells10102533

**Published:** 2021-09-24

**Authors:** Annoor Awadasseid, Yanling Wu, Wen Zhang

**Affiliations:** 1 Lab of Chemical Biology and Molecular Drug Design, College of Pharmaceutical Science, Zhejiang University of Technology, Hangzhou 310014, China; annoormohammed@ymail.com; 2 Institute of Drug Development & Chemical Biology, Zhejiang University of Technology, Hangzhou 310014, China; 3 Department of Biochemistry & Food Sciences, University of Kordofan, El-Obeid 51111, Sudan; 4 Lab of Molecular Immunology, Virus Inspection Department, Zhejiang Provincial Center for Disease Control and Prevention, Hangzhou 310051, China

**Keywords:** extracellular vesicles, exosomes, immunosuppression, chronic inflammatory, cancer

## Abstract

Exosomes are extracellular vesicles released by most of the eukaryotic cells. Exosomes’ components include proteins, lipids, microRNA, circular RNA, long noncoding RNA, DNA, etc. Exosomes may carry both pro and anti-inflammatory cargos; however, exosomes are predominantly filled with immunosuppressive cargos such as enzymes and microRNAs in chronic inflammation. Exosomes have surfaced as essential participants in physiological and pathological intercellular communication. Exosomes may prevent or promote the formation of an aggressive tumor and chronic inflammatory microenvironments, thus influencing tumor and chronic inflammatory progression as well as clinical prognosis. Exosomes, which transmit many signals that may either enhance or constrain immunosuppression of lymphoid and myeloid cell populations in tumors, are increasingly becoming recognized as significant mediators of immune regulation in cancer. In this review, we outline the function of exosomes as mediators of immunosuppression in tumor and chronic inflammatory microenvironments, with the aim to improve cancer therapy.

## 1. Introduction

Cancer is a severe illness that poses a significant threat to human health across the world [1,2]. Cancer is a hereditary illness on a molecular level, and the aberrant expression of tumor silencers and oncogenes is strongly connected to its progression [3]. Exosomes are a subgroup of extracellular vesicles (EVs) enveloped by a lipid bilayer membrane and released by most eukaryotic cells. They vary in size from 40 to less than 150 nanometers in diameter [4]. Exosomes were first discovered in the late 1980s and were thought to be only cellular waste products [5]. Scientists have now understood that exosomes constitute a new mechanism of intercellular communication and participate in various biological processes in health and disease, such as cancer, due to the rapid development of research methodology and procedures [6]. Exosomes’ biological activity depends on the bioactive cargos they carry, including lipids, metabolites, proteins, and nucleic acids that may be transferred to target cells [7,8,9,10]. Tumor-derived ***exosomes*** (TEXs), according to mounting evidence, play a vital role in cancer. Exosomes and their cargos might be used as a cancer prognostic marker, therapeutic target, or even a medication carrier for anticancer drugs [11].

**Figure 1:**
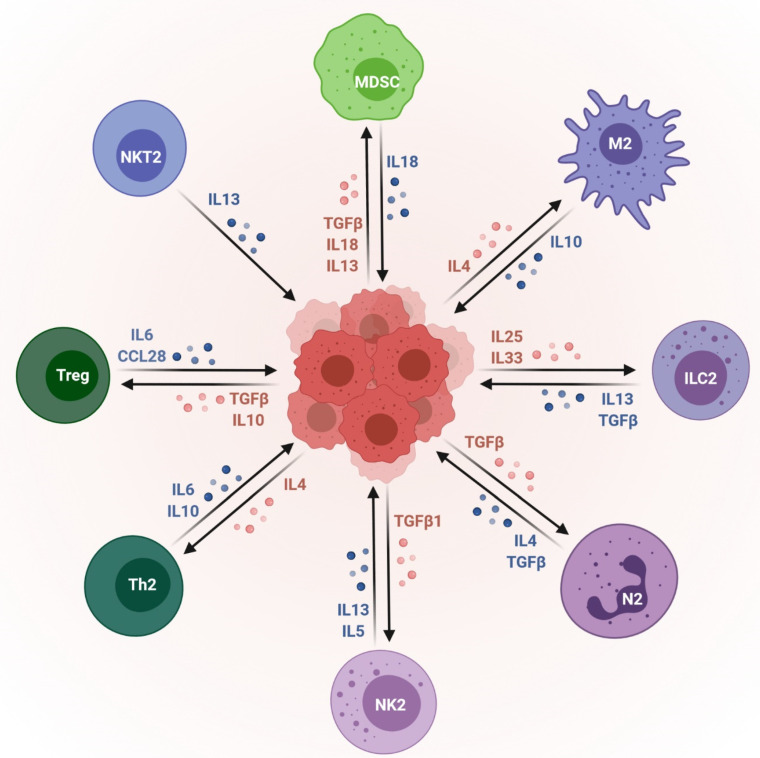
Immunosuppressive cells in the tumor microenvironment. The immunosuppressive component of the tumor microenvironment’s bidirectional interactions with tumor cells. Myeloid-derived suppressor cells (MDSC); anti-inflammatory macrophages (M2); group 2 innate lymphoid cells (ILC2); neuro-2 (N2); natural killer 2 (NK2); type 2 T helper (Th2); regulatory T cells (Treg); type 2 natural killer T (NKT2). This image was created using BioRender (http://biorender.com/ accessed on 10 August 2021).

Exosomes with immunosuppressive cargos are secreted by cells in chronic inflammation to counteract the detrimental immunological consequences caused by persistent inflammation. Exosomal signaling is used by cancer cells to increase the immunosuppressive microenvironment inside tumor sites (Figure 1) [12]. Immune detection and clearance of cancer cells are hampered by increased immunosuppression in tumors. Exosomes, which contain molecules that suppress inflammatory responses and promote regeneration, are secreted by mesenchymal stem cells and delivered to distant destinations of the body through the circulatory system [13]. Numerous soluble molecules, including miRNAs, enzymes, and other immunosuppressive factors, and several membrane-bound compounds capable of increasing immunosuppression in target cells, are found in the cargos of immunosuppressive exosomes [12].

The inflammatory response is a process that is triggered in response to tissue injury or pathogen identification, and it is aided by the innate and adaptive immune systems’ different cells and soluble mediators [14,15]. Inflammation will be eliminated in the normal physiological setting after the homeostatic state has been reached, either by eliminating the foreign pathogen or by effective tissue repair [16]. On the other hand, chronic inflammation may arise from uncontrolled inflammation that is not adequately treated and is associated with an increased risk of malignant cell transformation and cancer [15,17]. Cytokines have been shown to enhance both the establishment of optimal growth factors inside the tumor microenvironment and the continuation of tumor growth during chronic inflammation [18,19,20]. Tumor cells and their surrounding microenvironment may generate molecules that affect monocyte recruitment, migration, differentiation, and function [21].

Tumor inflammation is vital in forming a protumorigenic microenvironment, which aids in the initiation, promotion, and progression of tumorigenesis. Exosomes may help accomplish this by suppressing the immune system and preventing excessive inflammation. Exosomes, for instance, may cause immunosuppression by inducing apoptosis in immune cells [22]. Exosomes produced by Epstein–Barr virus (EBV) infected nasopharyngeal cells contain incredibly high levels of galectin-9 protein, which may cause apoptosis in mature Th1 lymphocytes [23,24]. Tumor-derived ***exosomes*** have also been shown to induce Fas-dependent apoptosis of activated CD8^+^ T lymphocytes in colorectal cancer and melanoma cells, allowing tumors to evade immunological detection [25,26,27].

Exosomes communicate with other cells by transferring their functional components to the target cells or engaging membrane-receptor-mediated signaling [28,29,30]. Preclinical research using exosomes for cancer therapy has shown promising findings from a translational standpoint [31,32,33]. Furthermore, exosomes generated from bodily fluids and circulation may be used as noninvasive liquid biopsies to identify diseases early and evaluate patient prognosis and response to treatment [30,34,35,36]. Exosomes have been found to contain proteins that protect them against phagocytosis and complement-mediated lysis, which could help explain why they are so stable [32,37]. The function of exosomes in influencing the immunological landscape of tumors is still being studied. The production of antigens by cancer cells kicks off an efficient antitumor immune response cycle, preceded by an antigen-presenting-cell (APC)-mediated processing and presentation of antigens to T lymphocytes. T lymphocytes that have been prepared to fight cancer enter the tumor and execute their antitumor activities [38]. Each step of this cycle is orchestrated by a wide range of cell-intrinsic and microenvironmental mechanisms that decide whether a protumor or antitumor immune response is established [38,39]. As a result, exosomes-derived signals in cancer may inhibit or enhance many components of immune responses [12,40,41,42,43]. Here, we discuss the current knowledge of exosomes as an immunotherapy for cancer treatment, the role of exosomes as immunosuppressive mediating variables in tumor and chronic inflammatory microenvironments, and future perspectives.

## 2. Exosomes as Immunotherapy for Cancer Treatment

**Figure 2:**
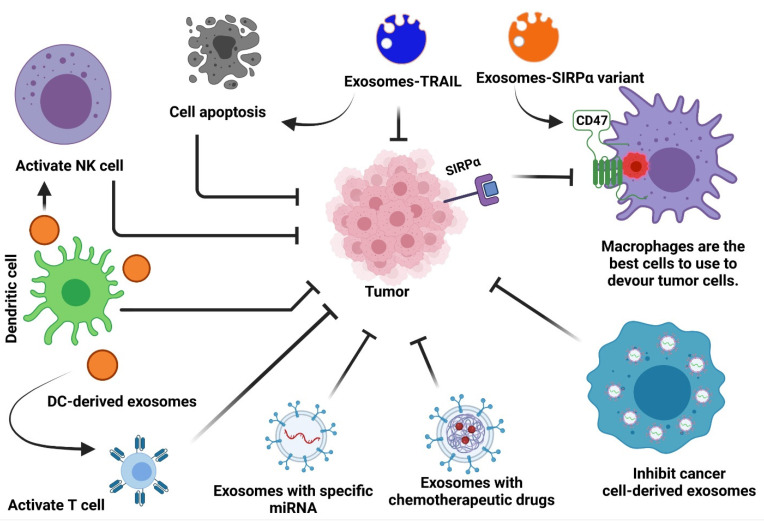
Exosome-based cancer therapies. Cancer treatments based on exosomes may be classified into two broad categories: 1. exosomes from immune cells repress cancer cells; 2. exosomes that serve as gene carriers.

Functional molecules may be found in exosomes obtained from many cell types, and this is believed to be what causes them to trigger immune responses in cancer. Exosomes were shown to be suitable for therapeutic delivery due to their biocompatibility, extended circulation half-life, and amenability to alteration [44,45,46,47]. They have been effectively utilized as delivery vehicles for nucleic acids, proteins, antibodies, nanobodies, compounds, and chemotherapeutic medicines, and excellent manufacturing techniques are sufficient to scale up their production [32,44,48,49,50,51,52,53,54,55,56,57,58]. Exosomes are nontoxic to mammals because they are usually produced in mammals, and they are not known to cause any significant problems in humans or mice [59,60,61,62]. Other processes, including phagocytosis, macropinocytosis, lipid-raft-mediated uptake, caveolin-mediated internalization, direct membrane fusion, and receptor-mediated entry, all effectively get exosomes into recipient cells [6,63]. Exosomes are a good vehicle for cancer immunotherapy because of these properties (Figure 2). The use of exosomes to engender tumor-associated antigens (TAAs) or immunostimulatory pathways in lymphoid and myeloid cells is beginning to develop since preclinical and clinical research has demonstrated its viability.

Compared to other intercellular communicators like cytokines, hormones, and neurotransmitters, exosomes offer a number of benefits [64]. While other mediators use different signals to activate a cell, exosomes are able to transmit several signals at once. A sialoglycoprotein-coated exosome may bind to the lymphocyte through adhesion molecules, then transport MHC and costimulatory chemicals. Moreover, exosomes may transmit several miRNAs to a cell at the same time, which allows them to reach a variety of mRNAs [64]. The function of exosomes as a natural nanocarrier for medication and gene delivery is extremely unique, considering that their ability to transmit intercellular communication is more varied than that of other gene and drug delivery methods. Exosomes are secreted by almost all cell types and are able to either target nearby cells or go to distant organs and tissues. Subsequently, exosomes are transferred into a new cell and liberate their payload to control protein expression and many signaling pathways [65,66,67]. Moreover, exosomes also carry surface cargo, including adhesion molecules, integrins, and tetraspanins, which may have a role in helping exosomes enter target cells by engaging with transmembrane receptors and ligands [68,69,70,71]. Exosomes may be taken up by recipient cells via endocytosis or penetrate the plasma membrane of those cells, therefore importing the proteins into the exosomal membrane [72]. Since exosomes may carry immunomodulatory drugs to particular target cells, they can be a helpful tool in fighting immunosuppression and stimulating anticancer immune responses.

Altered exosomes targeted T cells to deploy activating signals. An application termed synthetic multivalent antibodies redirected and activated T lymphocytes toward cancer cells, using exosomes as a delivery mechanism [51,73]. Through synthetic multivalent antibodies retargeted exosome (SMART-Exo), it was possible to activate and guide T cells to target EGFR- or HER2-expressing breast cancer cells. In vitro and in vivo, the SMART-Exo treatment enhanced antitumor immune responses [51,73]. B7-1 (CD80), B7-2 (CD86), or both were incorporated into leukemia cells, producing exosomes that were able to present B7-1 (CD80) and B7-2 (CD86) on their surfaces. L1210 cell challenge activated the B7 costimulatory system and T cells, increasing their proliferation and production of T_H_1 cytokines, with the result that L1210-inoculated mice developed protective immunity [74]. An investigational cell-free vaccine made of tumor peptide-pulsed DC-derived exosomes was used to treat cancer and has shown promising outcomes in the preclinical setting [75]. Despite demonstrating little therapeutic efficacy in phase I and II clinical study, this promising experimental treatment showed good safety and tolerability [60,61,62,76]. For a comprehensive list of clinical trials in cancer using DC-derived exosomes, see the published literature [77,78,79]. More research is required to substantiate its effect on cancer.

When the CD40 ligand activates the CD40 receptor, it stimulates the development of DCs and maturation, which leads to increased production of cytokines that help boost the immune system [80]. The 3LL Lewis lung cells were modified to generate exosomes with CD40L, which were then injected into other 3LL Lewis lung cells (CD40L-EXO). Activation of bone-marrow-derived DCs enhanced by CD40L-EXO was seen in vitro. Immunized animals that had splenocytes from which the CD40L-EXO plasmid had been extracted had greater production of T_H_1 cytokines, as well as improved cytotoxic activity in destroying 3LL cells, but not in destroying B16 cells. Tumor burden was reduced, and survival was extended in comparison to controls, in mice that had been vaccinated with CD40L-EXO and then challenged with 3LL tumors. Finally, CD40L-EXO caused antitumor responses in the tumors that had already developed [81]. Cancer-cell-derived exosomes were used to stimulate DCs, in combination with a TLR4 adjuvant signal, to improve the effectiveness of DC-based vaccines. As a result, the N-termi1 (N1ND) domain of HMGN1 was covalently linked to exosomes. DC_TEX-N1ND_-treated DCs, which have been designed to express exosomes, caused T cells to become more active and improved their ability to destroy cancer cells. In subcutaneous HCC, DC_TEX-N1ND_ slowed the development of the tumor and stimulated the immune system to destroy it. With DC_TEX-N1ND_ used as a therapy in orthotopic hepatocellular carcinoma, the tumor mass was reduced, as was the patient’s survival, and metastasis to the lungs was eliminated. This resulted in massive and robust antitumor immunity, which is substantial because of the import of DC_TEX-N1ND_ in creating effector and memory T cell populations, creating a large and sustained response [33].

An Exosome-based prostate cancer therapy vaccine was developed using a protein anchoring method that uses streptavidin-tagged IFN-γ immobilized at the cell membrane of biotinylated exosomes. This strategy builds on the hypothesis that increases in antigen presentation and maturation of APCs are both required to ensure full immune activation. Immunotherapy with the M1 macrophage polarization vaccine increased M1 macrophage polarization and allowed the cells to accumulate cancer-exposed immunosuppressive exosomes, resulting in greater tumor phagocytosis. The injection of the vaccine exosomes that target the RM-1 tumor in subcutaneous tumors delayed tumor development and extended life. Immunization also stimulated an increase in circulating immune cells with IFN-γ^+^ CD8^+^ T-cell receptors and a reduction in T-regulatory cells. In the end, the exosome-based vaccination triggered an immunological response in addition to an anticancer response [82]. TNF-α-mediated inflammation was also antagonized using exosomes, which serve as decoys that present the TNF-receptor-1-binding domain on their surface. Exosomes designed to block TNF-α have been shown to be effective in vitro [83]. In addition to their ability to provoke anti-cancer immune responses, scientists are now beginning to experiment with exosomes to lower or regulate immune system activity that often occurs in the context of autoimmune disorders.

## 3. Exosomes as Immunosuppressive Mediating Variables in Tumor Microenvironments

**Figure 3:**
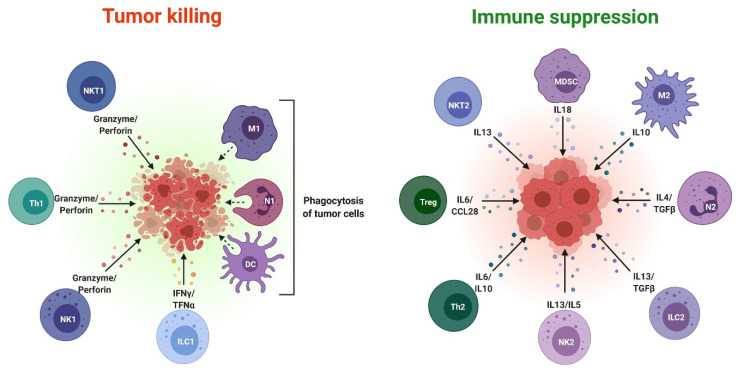
Pro and antitumor immune cells in the tumor microenvironment. Pro and antitumor effects of immune cell subsets in the tumor microenvironment, including common mediating cytokines. Proinflammatory macrophages (M1); neuro-1 (N1); dendritic cells; group 1 innate lymphoid cells (ILC1); natural killer 1 (NK1); type 1 T helper (Th1); type 1 natural killer T (NKT1). This image was created using BioRender (http://biorender.com/ accessed on 10 August 2021).

Chronic inflammation slows tumor development by creating an immunosuppressive microenvironment inside the tumor site by attracting and promoting the proliferation of immunosuppressive cells such as myeloid-derived suppressor cells (MDSCs) and regulatory T cells (Tregs) [84]. Immunosuppression is a strategy used by tumor cells to avoid immune detection. According to recent research, exosomes produced by tumor cells have played a key role in creating an immunosuppressive microenvironment in tumors [12]. For example, tissue-resident macrophages and infiltrating monocytes-macrophages are polarized towards the anti-inflammatory M2 phenotype by tumor-derived ***exosomes*** [85,86]. According to a previous study, in breast cancer, tumor-associated mesenchymal stem cells converted myeloid cells, such as monocytic MDSCs (M-MDSCs), into immunosuppressive M2 macrophages through exosomes. Tumor-derived ***exosomes*** may also promote the growth and activation of immunosuppressive cells in the tumor microenvironment, such as MDSCs, Tregs, and regulatory B cells (Bregs) [12,87,88]. On the other hand, exosomes may directly inhibit the activity of effector T cells, dendritic cells, and natural killer cells (NK cells) (Figure 3) [12].

Exosomal signaling not only controls the immunosuppressive phenotypes of immune cells at tumor sites, but immune cells also produce immunosuppressive exosomes. Exosomes generated from polymorphonuclear MDSCs (PMN-MDSCs), for example, may affect Th1 and Th17 cell activities [89]. Compared to parental MDSCs, tumor MDSCs exosomes exhibited an enhanced quantity of immunosuppressive molecules such as transforming growth factor-beta (TGF-β), miR-146a, and S100A [90]. Exosomes from PMN-MDSCs have previously been shown to enhance tumorigenesis by increasing the stemness and proliferation of colorectal cancer cells [91]. Treg cells may also use exosomes to inhibit effector immune cell activities, such as converting dendritic cells to the immunosuppressive tolerogenic state [92]. Remarkably, tumor-derived immunosuppressive ***exosomes*** have been shown to rise in cancer patients’ circulation. Several investigations have shown that the serum level of exosomal programmed death-ligand 1 (PD-L1), a great immunosuppressor, rises in cancer patients and is linked to tumor development [93]. This has a major impact on the body’s immune system; for example, PD-L1-based antibody treatments in cancer patients may be disrupted.

Exosomes, even those produced by the same cells, are highly diverse vesicles. Exosomes include immunosuppressive components comparable to those found in immunosuppressive cells such as MDSCs, Tregs, tolerogenic dendritic cells (tolDCs), and M2 macrophages, according to the characterization of exosomal cargos from the tumor microenvironment. Nevertheless, numerous nonimmune cell types, such as mesenchymal stem cells and cardiac endothelial cells, may produce immunosuppressive exosomes [88,94,95]. PD-L1 and cytotoxic T-lymphocyte-associated protein 4 (CTLA-4), arginase 1 (ARG1), high-mobility group box 1 protein (HMGB1), and TGF-β are among the proteins found in tumor cell exosomes related to immunosuppression [30,96,97,98,99]. For example, PD-L1 binds to effector T cells’ programmed cell death protein 1 (PD-1) receptor and suppresses their immunological function. Immunosuppression is triggered by the proteins ARG1 and TGF-β. Furthermore, certain apoptotic inducers, including tumor-necrosis-factor-related apoptosis-inducing ligand (TRAIL) and Fas ligand (FasL), as well as toll-like receptor 2 (TLR2), may be found in exosomes [100,101].

CD39 and CD73 proteins, which are ectonucleotidases, may also be found on the exosomal membrane [102]. CD39 and CD73 transform adenosine triphosphate/adenosine diphosphate (ATP/ADP) nucleotides produced by stressed cells to adenine, a strong immunomodulators that increases immunosuppression [103]. Exosomes often lack key signaling molecules such as the STAT3 and FoxP3 proteins. On the other hand, exosomes include many miRNAs, which control both the signaling and transcription of important immunosuppressive components [104]. Many miRNAs may cause MDSCs or Tregs to expand. Furthermore, some miRNAs may help macrophages become more M2 polarized [104]. Because miRNAs have multiple target mRNAs utilized in a context-dependent way, it isn’t easy to evaluate their importance in age-related immunosuppression.

## 4. Exosomes as Immunosuppressive Mediating Variables in Chronic Inflammatory Microenvironments

Inflamed tissue releases large amounts of stress and therefore causes an increase in the release of exosomes [105,106]. While exosomes are capable of provoking both proinflammatory and anti-inflammatory responses, they can only trigger one response type depending on the context and other aspects. While dealing with acute traumas, such as tissue injury and hypoxia, proinflammatory exosomes are released; when dealing with chronic inflammatory situations, immunosuppressive exosomes are released from both immune and nonimmune cells. Immune cells produce proinflammatory exosomes in the immediate postinjury period after an episode of cerebral ischemia and kidney damage [107,108]. Anti-inflammatory exosomes also seem to be present in inflammation-promoting illnesses such as arthritis, atherosclerosis, and sepsis, and they seem to possess both an anti-inflammatory and proinflammatory character [106]. Another example of this concept is found in a study in which researchers injected an intravenous bolus of the exosomes that had been isolated from mouse serum of lipopolysaccharide-exposed septic shock or a high-fat diet into the brains of mice. The researchers found that these exosomes activated microglia and led to an increase in neuroinflammation [109]. Many inflammatory miRNAs, including miR-15a, miR-27b, and miR-125a, were found in these exosomes. In systemic inflammation, some cellular debris is released in the form of exosomes, which may move through the bloodstream and spread inflammation to distant organs.

Although chronic inflammatory diseases seem to be linked with exosomes that carry anti-inflammatory and immunosuppressive cargos, the data are not entirely conclusive [105,106]. Immunosuppressive cells (MDSCs and Tregs) are recruited into inflamed tissues when there is a delay in resolving the inflammatory process. Finally, resident immune cells such as macrophages, microglia, and dendritic cells are induced to take on an immunosuppressive phenotype. That is, they move from the M1 state, which is characterized by an inflammatory response, to the M2 state, which is characterized by an anti-inflammatory response [110]. Exosomes are used alongside conventional methods as well as those involving immunosuppressive cells in an effort to inhibit immunological activities. Several immune cells, including exosomes-induced Tregs, can take up exosomes produced by mice MDSCs [111]. Th cells are suppressed, and MDSC-derived exosomes lower the cytotoxicity of T cells. MDSCs carrying miR-29a and miR-93 reduced the incidence of collagen-induced arthritis in mice, according to the findings of Zhu et al. [89]. Okoye et al. showed that Treg-derived exosomes inhibit the proliferation and cytokine release from Th1 cells by decreasing the production of Let-7d miRNA [112]. Dendritic cells (also known as monocyte-derived exosomes) are a vital part of the Treg-derived exosomes. One such example is from Tung et al., who found that Tregs secrete miR-142 and miR-150, which then cause dendritic cells (DCs) to become tolerogenic (tolDCs). Increased production of anti-inflammatory IL-10, but not proinflammatory cytokines, were seen after tolDCs treatment with lipopolysaccharide [92]. The study also discovered that M2 macrophages and microglia also release immunosuppressive exosomes. For example, Kim et al. found that M2 macrophage-derived exosomes could convert proinflammatory M1 macrophages into M2 phenotype macrophages that favored cutaneous wound healing in mice. The M2 macrophages in which the reprogramming process had occurred highly produced ARG1, IL-10, and TGF-β, and they had a major impact on angiogenesis and tissue healing [113]. Additionally, Song et al. found that M2 microglia-secreted exosomes reduced ischemia-reperfusion injury in the mouse brain and improved neural survival. This favorable impact was mostly due to the exosomal mir-124 miRNA, which was produced within the cancer cells [114]. While bound to an antigen by the antigen-presenting cells, such as dendritic cells, macrophages, and B cells, exosomes secrete MHCI and MHCII proteins [105]. Exosomes loaded with peptide/MHC complexes deliver these complexes to immune cells, such as T cells. In a context-dependent manner, exosomal MHCs appear to have the ability to inhibit the body’s immune response. Most of the immune cells found in these investigations secrete exosomes, which serve as mobile systems to transport immune-suppressive substances into the target cells to reduce inflammation.

**Figure 4:**
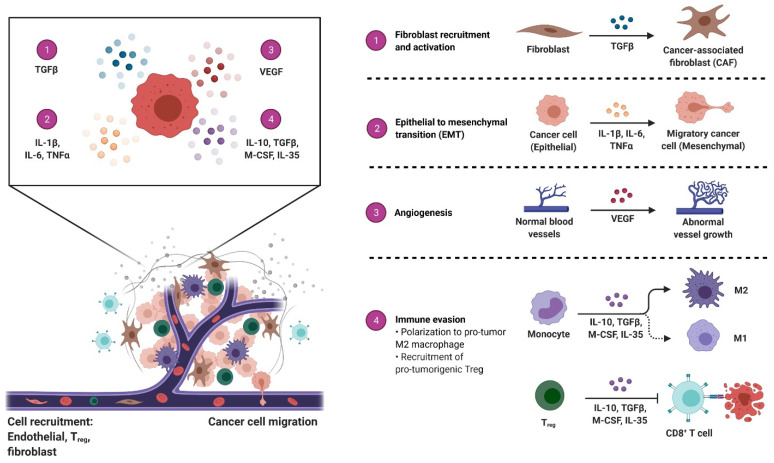
The tumor microenvironment: overview of cancer-associated changes. The tumor microenvironment (TME) includes the cellular components surrounding the tumor mass, such as immune cells, fibroblasts, and epithelial cells, and the acellular components, including the extracellular matrix and blood vessels. Cancer cells release molecules that modulate the TME and contribute to cancer growth through immune evasion, metastatic niche formation, and neoangiogenesis, among other functions that contribute to the hallmarks of cancer. This image was created using BioRender (http://biorender.com/ accessed on 10 August 2021).

In addition to being produced by immune cells, exosomes that have immunosuppressive properties are also released by nonimmune cells, particularly tumor cells and mesenchymal stem cells. One particularly fascinating example of this is a recent study that showed that B cells generated from mouse cardiac endothelial cells could produce immunosuppressive regulatory B cells (Bregs) [95]. Yu et al. discovered that endothelial cells produce exosomes that reduce inflammation and apoptosis caused by hypoxia-reperfusion. The exosomes that were found to contain miR-199a lowered endoplasmic reticulum stress, protecting neural cells from the potential effects of the stress. This seems to show that secretion of exosomes from immune or nonimmune cells may reduce inflammation while simultaneously promoting tissue repair/regeneration [115]. Borges et al. showed that tubular epithelial cells of mouse kidneys produced hypoxia-evoked exosomes that led to the activation of tissue fibroblasts, resulting in regeneration and fibrosis. The TGF-β mRNA present in these exosomes triggered the activation of fibroblasts, resulting in fibrosis (Figure 4) [116]. As mentioned above, it has been scientifically shown that mesenchymal stem cells (MSC) are superior to other stem cell sources when it comes to producing immunosuppressive and regenerative exosomes [13]. Multipotent stromal cells are often found in the bone marrow and adipose tissue, both of which are places where multipotent stromal cells may be found. Although MSC-derived exosomes are now being investigated as a possible treatment for a number of autoimmune and inflammatory disorders, their use in clinical practice is currently under investigation [13]. Exosomes produced by MSCs are known to carry immunosuppressive miRNAs and proteins that circulate to inflamed tissues and do their work in the bloodstream. Microvascular-derived exosomes produce immune-modulatory phenotypes, such as M2 macrophages, tolDCs, and Tregs, among others. One example of how MSCs from the bone marrow improve inflammatory responses and the severity of a traumatic brain injury in mice is that exosomes they release may help to limit inflammation and speed recovery [117]. Researchers have shown that circulating MSC-derived exosomes may consistently be found in several afflicted organs for therapeutic investigations. Immunosuppressive characteristics such as treating rat spinal cord damage, rat rheumatoid arthritis, mouse autoimmune hepatitis, and mouse chronic airway inflammation have been found in MSC-derived exosomes [118,119,120,121]. Domenis et al. also found that cytokine-treated human adipose MSCs produced proinflammatory cytokines, which subsequently enhanced the immunosuppressive capacity of exosomes generated from these MSCs, e.g., they promoted the M2 polarization of macrophages [122]. The fact that the inflammatory mediators here influence myeloid cell differentiation, e.g., MDSCs and Tregs, but also boost the immunosuppressive activity of MSC-derived exosomes suggests that these mediators may have significant impacts on the process of myeloid cell differentiation.

## 5. Future Perspectives

Based on a considerable amount of data, it has been concluded that exosomes may affect different immune cells in both an active and a suppressor manner. Exosomes have characteristics that enable them to move and transfer their cargo. This implies they may be used as an efficient way to cure cancer and treat other diseases via immunotherapy. Exosome-mediated loading of tumor-associated antigens and adjuvants is thought to trigger a significant antigen-specific immunostimulatory effect [45]. In the experiment described by Morishita et al., a tumor-derived ***exosome*** containing endogenous tumor antigen and streptavidin lactadherin was engineered to be coupled with biotinylated CpG DNA, allowing the injection of biotinylated CpG DNA into the mice bearing the tumor. The tumor-bearing animals were administered exosomes containing the foreign DNA of the murine melanoma B16-BL6 tumor, which had an in vivo antitumor impact [123]. Mahmoodzadeh et al. found that staphylococcal enterotoxin B (SEB) is encapsulated inside cancer cell exosomes, which activates T lymphocytes by binding to MHCII. Treated ER-negative breast cancer cells were found to suffer substantial apoptosis after treatment with these exosomes [124]. Additionally, it has been shown that exosome loading with miRNAs can also provoke immunological responses in the cells they are delivered to. In the experiment by Momem-Heravi et al., it was demonstrated that exosomes loaded with miR-155 were able to enter macrophages in vitro and in vivo, activate and differentiate these cells into an M1-like phenotype, and elicit an inflammatory response [125]. Although now there are many ways to load exosomes with cargo, including electroporation, sonication, direct transfection, and simple incubation, at the moment, just a few of them are in common use [44]. Yet, more research is needed to enhance the potential of exosomes as an immunotherapy option.

Another method of promoting immune response for cancer treatment is dendritic-cell-derived exosome therapy [126]. The treatment provides a process to encourage dendritic cells (DCs) to release exosomes that contain the appropriate antigens. The DC-derived exosomes are isolated, purified, and used to provoke an immune response in a few different ways. One method involves presenting the exosomes to T cells to activate them directly, whereas the other method utilizes the transfer of the antigen-MHC complex to DCs to induce an immune response [127,128,129]. According to clinical studies, two phase I human clinical trials performed on melanoma, and nonsmall cell lung cancer found that immune-stimulatory effects from DC-derived exosomes have shown encouraging outcomes, but only moderate results [60,61]. Additionally, phase II clinical trial results in advanced nonsmall cell lung cancer (NSCLC) patients who have received chemotherapy shown that DC-derived exosomes do not enhance the progression-free survival of patients when they are used in the first stages of treatment [76]. Although it is still in the developmental stages, the use of exosomes as a possible form of cancer immunotherapy has yet to be realized. Further, numerous obstacles still exist. The variety of functions played by exosomes in cancer and immunomodulation, as well as the details of the logistics involved in developing exosomes as immunotherapy, must be investigated further. There is further research on the effectiveness of isolation methods for exosomes, as well as the potential of off-target effects owing to the transmission of unknown components in exosomes [64,67].

## 6. Conclusions

One of the major hurdles with emerging medication possibilities, such as proteins and nucleic acids, is that they tend to be unstable in vivo, making the drugs’ effectiveness even more uncertain. Many delivery methods for nanoparticles are problematic; therefore, exosomes as a mimic of “nature’s delivery systems” are useful for delivering the biological molecules they carry. Due to their tiny size and natural cellular makeup, these vehicles may escape phagocytosis or destruction by macrophages, and they can circulate throughout the body for long periods. Compared to the liposomes and polymeric nanoparticles that are common in nanoparticulate systems, exosomes bypass the endosomal pathway and lysosomal breakdown while also allowing the transport of cargos directly into the cytoplasm. By bypassing the endosomal pathway, the effectiveness of siRNA transfection is improved [130]. Due to the exosomes’ nature, they have intrinsic stability and are already targeted. One benefit of these medication delivery vehicles is their ability to traverse the blood–brain barrier [64,131]. It is challenging to determine exosomes’ long-term safety and efficacy since the knowledge of their nature and function is incomplete. A more comprehensive understanding of in vivo trafficking, biological destiny, and its effect on the targeted organs is needed. Various difficulties exist in understanding exosomes, including drug cargo loading and delivery assembly.

There is presently no one best method of exosome purification for isolating highly pure exosomes [132]. It is more difficult to produce a significant amount of exosomes from the isolation procedures, and exosome manufacturing for clinical trials and postapproval research is costly [133]. Future clinical usage of exosomes is likely to include a hybrid kind of exosome design [71], and combining exosomes with therapeutic cargos may lead to negative consequences. For such systems to be developed, the safety and effectiveness parameters must be fully described. The chemical conjugation of functionalized exosomes’ ligands to their surface must be studied. This will allow for an investigation of the functionalized exosome and targeting molecular combinational products and techniques. An increasing number of therapeutic cargo and functioning exosomes or hybrid exosome-mimics integrative investigations are considered necessary. Even though much biological knowledge is currently available, exosomes contain disparate parts and may present immunological effects depending on the type of the parent cells. The issue of exosomes playing a role in tumor development or aiding in the release of membrane antigens that facilitate tumor growth is significant. Additionally, exosomes with caspase-3 may also stop cells from dying by apoptosis or make tumor cells more resistant to chemotherapy by blocking chemotherapeutic drug accumulation [134]. An option to combat the aforementioned problems is to create artificial exosomes or exosome mimetics that can avoid triggering an adverse immune response [135].

The interest in exosomes is growing rapidly because of their highly significant heterogeneity and potential to alter the tumor’s immune microenvironment. In addition to participating in interactions between different cells, immune cells also use intercellular exosomal signals to govern immune activities. As of this moment, it is not completely understood how the cargo of exosomes and the activation of exosomal proteins are regulated. The use of exosomes for the diagnosis and prognosis of illnesses and their usage as therapeutic vehicles also face unique difficulties [136]. There is a strong relationship between inflammatory states and exosomal signaling. Notably, persistent inflammation primes the differentiation of many kinds of immunosuppressive exosomes. Thus, inflammation-mediated factors may also trigger the migration of stem cells beyond the vasculature, such as bone marrow mesenchymal stem cells. Currently, they are a promising treatment model for autoimmune and inflammatory illnesses since exosomes from mesenchymal stem cells have significant immunosuppressive activity. Although many studies have attempted to characterize the molecular process of how exosomes are generated, the investigation into endocytosis and exosome biology is still required to improve exosomes’ diagnostic and therapeutic capacities. However, the exosomes used in the in vitro systems often have greater concentrations than in the human body, so it’s important to keep in mind that these studies may have limited results. We anticipate that, with ongoing study, we may utilize exosomes as natural carriers to take advantage of their benefits while bypassing their limitations. New cancer therapy methods will be developed using exosomes to benefit vast numbers of cancer patients.

## Data Availability

Not applicable.
